# Mr. George Oldham Liddall

**Published:** 1910-06-11

**Authors:** 


### OBITUARY.

The death is announced of Mr. George Oldham Liddall, at the age of 75. We understand that he was an M.R.C.S. and an L.S.A., and that he served in the Baltic in 1885 on H.M.S. *Argonaut.*

